# Myosin-V Induces Cargo Immobilization and Clustering at the Axon Initial Segment

**DOI:** 10.3389/fncel.2017.00260

**Published:** 2017-08-28

**Authors:** Anne F. J. Janssen, Roderick P. Tas, Petra van Bergeijk, Rosalie Oost, Casper C. Hoogenraad, Lukas C. Kapitein

**Affiliations:** Cell Biology, Department of Biology, Faculty of Science, Utrecht University Utrecht, Netherlands

**Keywords:** polarized transport, motor proteins, myosin-V, kinesin, axon initial segment, motor cooperation

## Abstract

The selective transport of different cargoes into axons and dendrites underlies the polarized organization of the neuron. Although it has become clear that the combined activity of different motors determines the destination and selectivity of transport, little is known about the mechanistic details of motor cooperation. For example, the exact role of myosin-V in opposing microtubule-based axon entries has remained unclear. Here we use two orthogonal chemically-induced heterodimerization systems to independently recruit different motors to cargoes. We find that recruiting myosin-V to kinesin-propelled cargoes at approximately equal numbers is sufficient to stall motility. Kinesin-driven cargoes entering the axon were arrested in the axon initial segment (AIS) upon myosin-V recruitment and accumulated in distinct actin-rich hotspots. Importantly, unlike proposed previously, myosin-V did not return these cargoes to the cell body, suggesting that additional mechanism are required to establish cargo retrieval from the AIS.

## Introduction

The selective transport of different cargoes into axons and dendrites underlies the polarized organization of the neuron. Recent work has revealed that different motor proteins have a different selectivity for axons and dendrites. For example, some kinesins selectively target axons, while others target both axons and dendrites (Huang and Banker, 2012; Lipka et al., 2016). In addition, myosin-V has been implicated in selective targeting to dendrites (Lewis et al., 2009). Expression of a dominant negative form of myosin-Va caused the non-specific localization of cargo otherwise enriched in the somatodendritic compartment. Furthermore, coupling a protein to a myosin-Va binding domain was sufficient to cause its somatodendritic localization. More recent work has reported that vesicles with dendritic cargoes often enter the axon, but stop and reverse in the axon initial segment (AIS) in a process that depends on myosin-Va and an intact actin cytoskeleton (Al-Bassam et al., 2012). Nevertheless, the exact contribution of actin and myosin-V to axonal exclusion has remained controversial, given that actin disruption also distorted the sorting of cargoes into the proper carriers (Petersen et al., 2014). In addition, whether recruitment or activation of myosin-V is sufficient to cause the reversal of dendritic cargo has remained unclear.

Although, it has become clear that the combined activity of different motors determines transport destination and selectivity, little is known about the mechanistic details of motor cooperation. For example, it is not known whether acute activation or recruitment of myosin-V is sufficient to oppose kinesin-based axon entries. More generally, how the outcome of multiple motors depends on the relative amounts of motor proteins recruited to cellular cargoes has remained unexplored. Elegant *in vitro* assays have used DNA origami to assemble well-defined combinations of different motor proteins (Derr et al., 2012), but similar control has not yet been achieved inside cells. Previously, acute recruitment of different motor proteins using chemically-induced heterodimerization has been used to probe combinatorial motor activity in non-neuronal cells (Kapitein et al., 2010b, 2013). These experiments revealed that, in non-neuronal COS7 cells, recruitment of myosin-V is sufficient to attenuate kinesin-propelled cargo (Kapitein et al., 2013). However, in these assays, the motors could not be recruited independently, which would enable sequential recruitment of different motors.

Here we introduce a new assay that allows the independent recruitment of different motor proteins. We find that recruiting myosin-V to kinesin-propelled cargoes at approximately equal numbers is sufficient to stall motility. Kinesin-driven cargoes entering the axon were arrested in the AIS upon myosin-V recruitment and accumulated in distinct actin-rich hotspots. Importantly, unlike proposed previously, myosin-V did not return these cargoes to the cell body, suggesting that additional mechanism are required to establish cargo retrieval from the AIS.

## Results

To establish an assay for the independent recruitment of different motors, the FKBP-rapalog-FRB heterodimerization system was combined with a recently introduced chemically-induced heterodimerization system in which the cell-permeable, AM-modified plant hormone gibberellin triggers the interaction between a GID1 and a GAI domain (Miyamoto et al., 2012). We chose this dimerization system over light-induced systems, because, similar to the FKBP-rapalog-FRB system, the induced complex formation has been reported to be essentially irreversible. As a result, the available sites on the cargoes will be quickly saturated as long as the number of GAI-labeled motors in the cell is higher than the total number of GID1-sites on the cargoes, irrespective of the exact concentrations. Therefore, if both heterodimerization systems are combined in one peroxisome-targeting construct, PEX3-mRFP-FKBP-linker-GID1, the FRB and GAI domains will be recruited with roughly equal probabilities.

**Figure 1:**
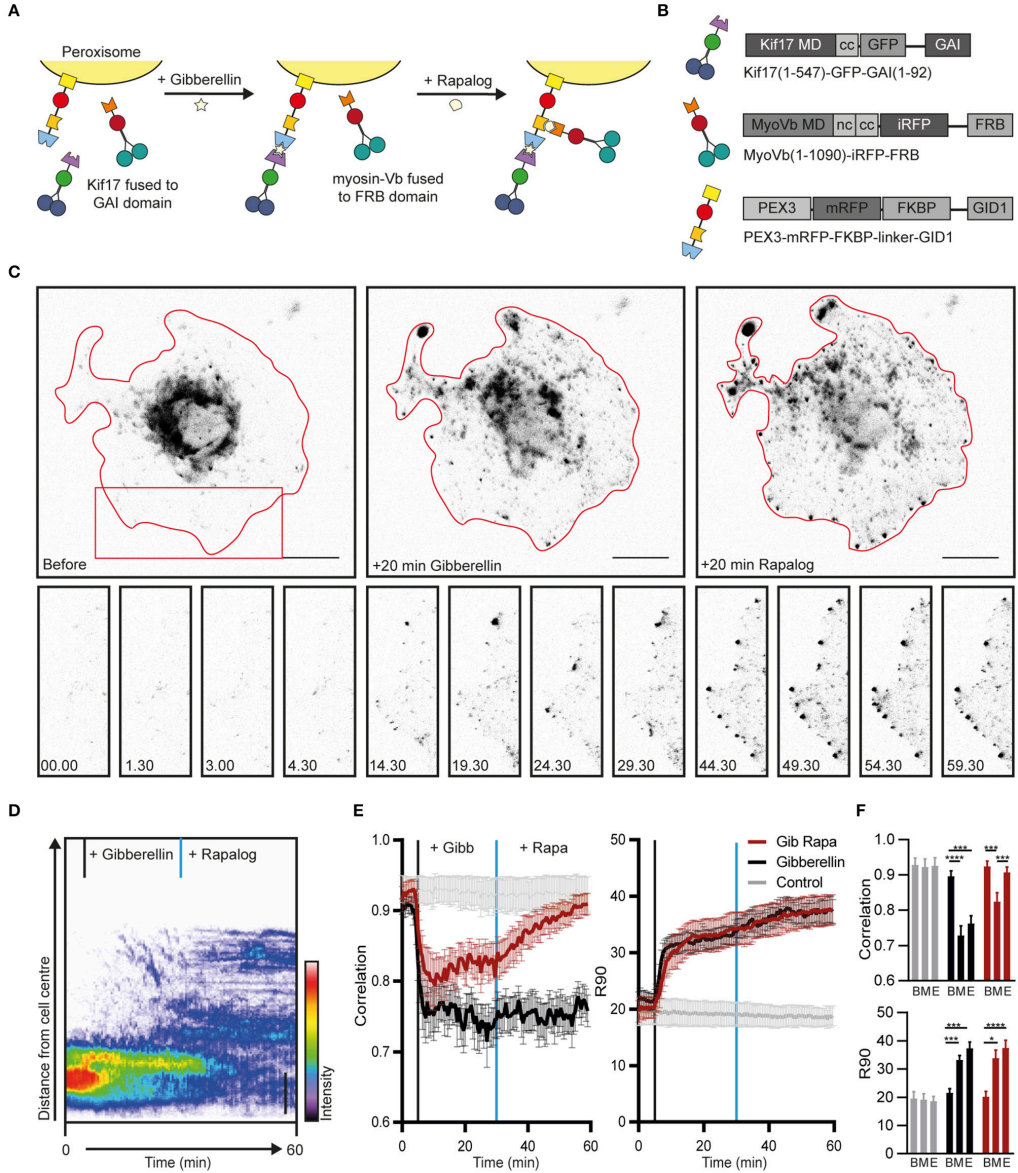
Sequential recruitment of kinesin and myosin-V in COS7 cells. **(A)** Assay: Sequential recruitment of kinesin and myosin-V by addition of gibberellin and rapalog, respectively. **(B)** Overview of constructs. MD, motor domain; CC, coiled coil; NC, neck coil; GAI, gibberellin insensitive; GID1, Gibberellin insensitive dwarf1. **(C)** Peroxisome distribution before recruitment of motors and after sequential recruitment of kinesin and myosin-V. Red curves indicate cell outline. Panels show individual frames of a cut out. Scale bar, 20 μm. **(D)** Radial kymograph indicating the redistribution of fluorescent peroxisomes relative to the cell axis. Vertical lines indicate addition of gibberellin (black) and rapalog (blue). Scale bar, 10 μm. **(E)** Displacement (expressed in R_90%_) and correlation (frame-to-frame similarity from 0 to 1) vs. time for cells without added ligands (control, gray), cells with addition of gibberelin only (Gibberelin, black), and cells with addition of gibberellin and rapalog (Gib + rapa, red). *N* = 12, 16, and 14 cells for control, gibbellin, and gib+rapa groups, respectively. Data was obtained from 2 experiments, mean ± s.e.m. Vertical lines indicate time of addition of gibberellin (black) and rapalog (blue), if added. **(F)** Averages of ten frames of each cell at *t* = 0–4.5 min (before, B), *t* = 25–29.5 min (middle, M), and *t* = 55–59.5 min (end, E). ^*^P < 0.05, ^***^P < 0.001, ^****^P < 0.0001, Friedman test, Dunn's *post-hoc* test.

To test the independent recruitment of different motors, COS7 cells were transfected with PEX3-mRFP-FKBP-linker-GID1, Kif17-GFP-GAI, and MyoVb-iRFP-FRB (Figures 1A,B). Upon addition of gibberellin, Kif17-GFP-GAI was recruited to PEX3-mRFP-FKBP-linker-GID1 and peroxisomes were rapidly redistributed to the cell periphery (Figure 1C, middle column). Similar to previous observations, peroxisomes in the cell periphery remained mobile even after reaching the periphery of the cell (Kapitein et al., 2013). In addition, contrary to FKBP-based heterodimerization, we noted that a subset of peroxisomes was already mobile at the periphery before addition of gibberellin, indicating some degree of background heterodimerization. To still ensure independent recruitment of different motors, gibberelin-based motor recruitment was always performed prior to rapalog-based recruitment.

Twenty five minutes after addition of gibberellin to recruit kinesin, rapalog was added to recruit myosin-V (Figure 1A), resulting in an arrest of the kinesin-driven motility and the accumulation of peroxisomes near the cell cortex (Figure 1C, right column, Figure 1D; Supplemental Video 1). These effects were quantified using two previously introduced metrics (van Bergeijk et al., 2015). First, we calculated for all frames the radius required to include 90% of the fluorescence intensity of the peroxisomes (R_90%_), which revealed that peroxisomes moved rapidly to the periphery upon recruitment of kinesin, but did not move much further upon recruitment of myosin-V (Figures 1E,F). Second, we used image correlation analysis to measure the overall frame-to-frame similarity during the experiment. In the absence of transport, two subsequent images are largely identical and the correlation index will be close to 1, whereas a value of 0 indicates that all organelles have moved to previously unoccupied positions. The correlation index decreased upon kinesin recruitment, reflecting the increased peroxisome mobility, whereas it increased after recruitment of myosin, indicating that peroxisome became less motile (Figures 1E,F). This decrease in motility was not observed without recruitment of myosin although cargo reached the cell periphery. This is reflected in the correlation index which remains low without myosin recruitment. Thus, different heterodimerization systems can be combined to independently recruit different motor proteins, and the recruitment of myosin-V to kinesin motors at roughly equal numbers is sufficient to arrest kinesin-driven motility.

**Figure 2:**
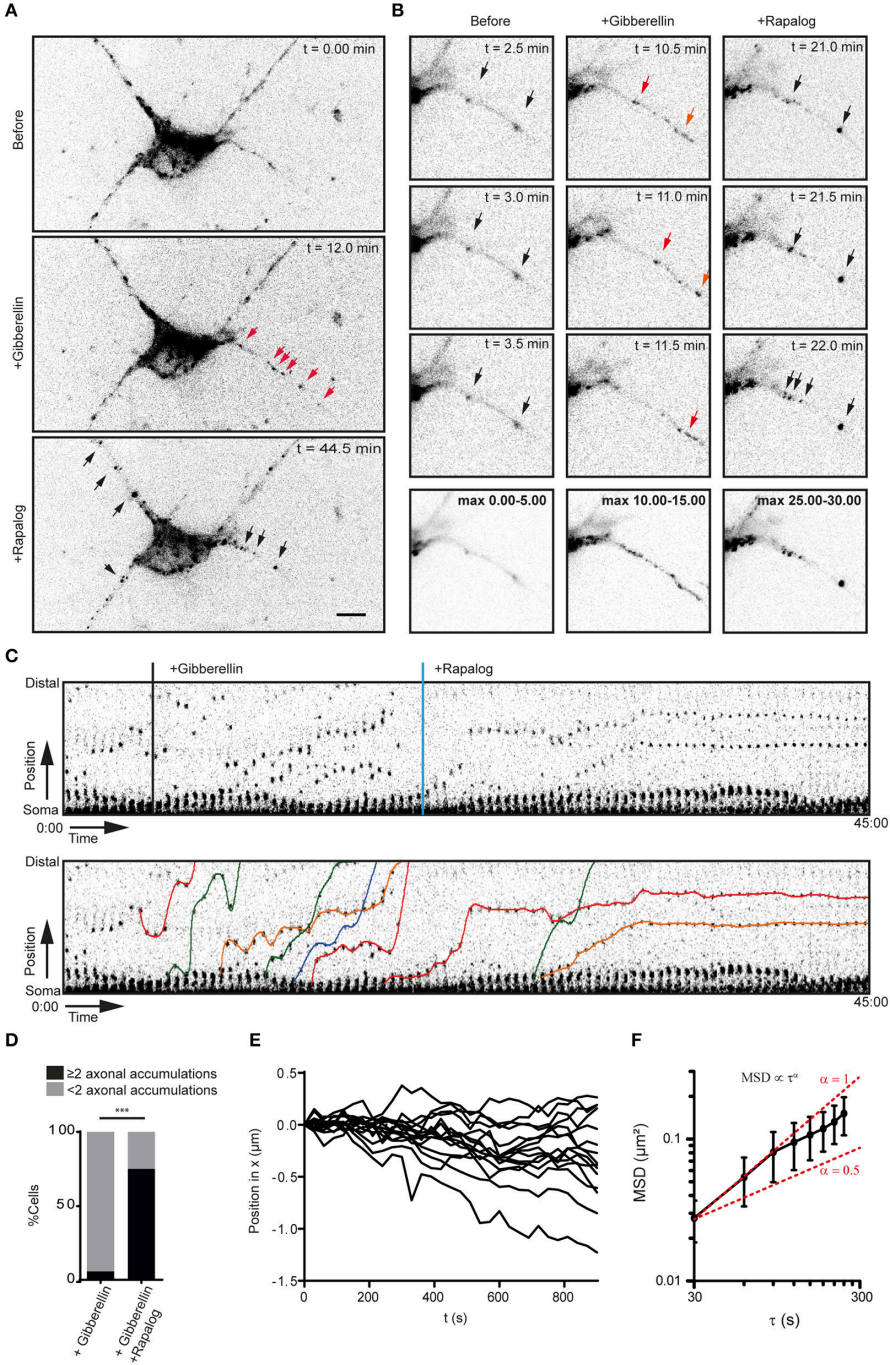
Myosin-V anchors kinesin-1 propelled peroxisomes in the proximal axon and somatodendritic compartment. **(A)** Dissociated hippocampal neuron showing the distribution of PEX3-mRFP-FKBP-linker-GID1 before (top), after the recruitment of Kif5-GFP-GAI through addition of gibberellin (middle) and after the addition of rapalog to recruit MyoVb-iRFP-FRB (bottom). Red arrows indicate motile peroxisomes, black arrows indicate non-motile peroxisome accumulations. Scale bar, 10 μm. **(B)** Zoom of the proximal axon of neuron in **(A)** before (left), plus gibberellin (middle), and plus rapalog (right). Bottom row shows a maximum projection of a 5 min interval before (left) or after the addition of dimerizers (middle, right). **(C)** Sequential frames of the proximal axon of a dissociated hippocampal neuron treated and imaged as in **(A)**. Manually annotated tracks are displayed superimposed on the bottom panel. **(D)** Number of stalled peroxisome accumulations in the axon before and after anchoring with MyoVb. In all cases, recruitment of kinesin upon addition of gibberellin induced a burst of perxosisomes into the axon. *n* = 16 and *n* = 24 for control and +Rapalog, respectively ^***^p < 0.001 Fisher Exact test. **(E)** Relative displacements of myosin-V anchored clusters 10 min after the addition of rapalog. Negative and positive displacement indicates retrograde and anterograde movement, respectively. **(F)** Mean square displacement analysis of myosin-V anchored peroxisome clusters tracked for at least 25 intervals of 30 s mean ± *sd* (*n* = 26). Red lines show example curves with slopes α = 1 and α = 0.5 (i.e., log(MSD) α log τ, representative of purely diffusive or confined motility, respectively.

We next switched to neurons to examine how activation of myosin-V alters the kinesin-driven transport of peroxisomes into the axon. We used the kinesin-1 KIF5B, because this motor has been reported to efficiently target cargoes selectively into the axon (Song et al., 2009; Kapitein et al., 2010a; Petersen et al., 2014; Supplemental Figures 1A–C). Indeed, the addition of gibberellin to dissociated hippocampal neurons expressing PEX3-mRFP-FKBP-linker-GID1, Kif5-GFP-GAI, and MyoVb-iRFP-FRB induced a burst of peroxisome motility into the axon (*n* = 16 cells, Figures 2A–D). Remarkably, the subsequent addition of rapalog to recruit myosin-V resulted in the appearance of several spots in the proximal axon where axon-entering peroxisomes would cluster together, whereas further down the axon (>35 μm) such clusters did not emerge (*n* = 15 cells, cells with ≥2 induced clusters and >75 μm axon length imaged were included, Figures 2A–D; Supplemental Video 2). Quantification revealed that the percentage of cells that had more than two proximal axonal accumulations increased from 6.25 to 75% during the first 40 min after addition of rapalog (Figure 2D). Thus, the acute, close-to-equimolar recruitment of myosin-V to kinesin-1 driven, axon-entering cargoes clusters these cargoes in the proximal axon.

Previous work has suggested that myosin-V can drive retrograde axonal transport, thereby returning to the cell body cargoes that have erroneously entered the axon (Watanabe et al., 2012). In contrast, we observed that myosin-V induced the appearance of cargo clusters that were largely immobile. To analyze the motility of the myosin-V induced peroxisome clusters in more detail and test for retrograde motility, we traced individual peroxisome clusters (Figure 2E) and averaged their mean-squared displacements (MSD) for different time intervals (Figure 2F). The power dependence α of the MSD with increasing time intervals τ, MSDα τ^α^, is the anomalous diffusion exponent (Saxton and Jacobson, 1997) and indicates whether motility is completely random (α ≈ 1, diffusive), directed (1 < α ≤ 2, superdiffusive), or confined (0 < α < 1, subdiffusive). Our analysis revealed that the clusters were confined and that the average displacement over >13 min was <500 nm [i.e., (0.25 μm^2^)^1/2^, Figure 2F]. Thus, myosin-V does not drive retrograde transport, but anchors cargo at specific locations in the proximal axon.

**Figure 3:**
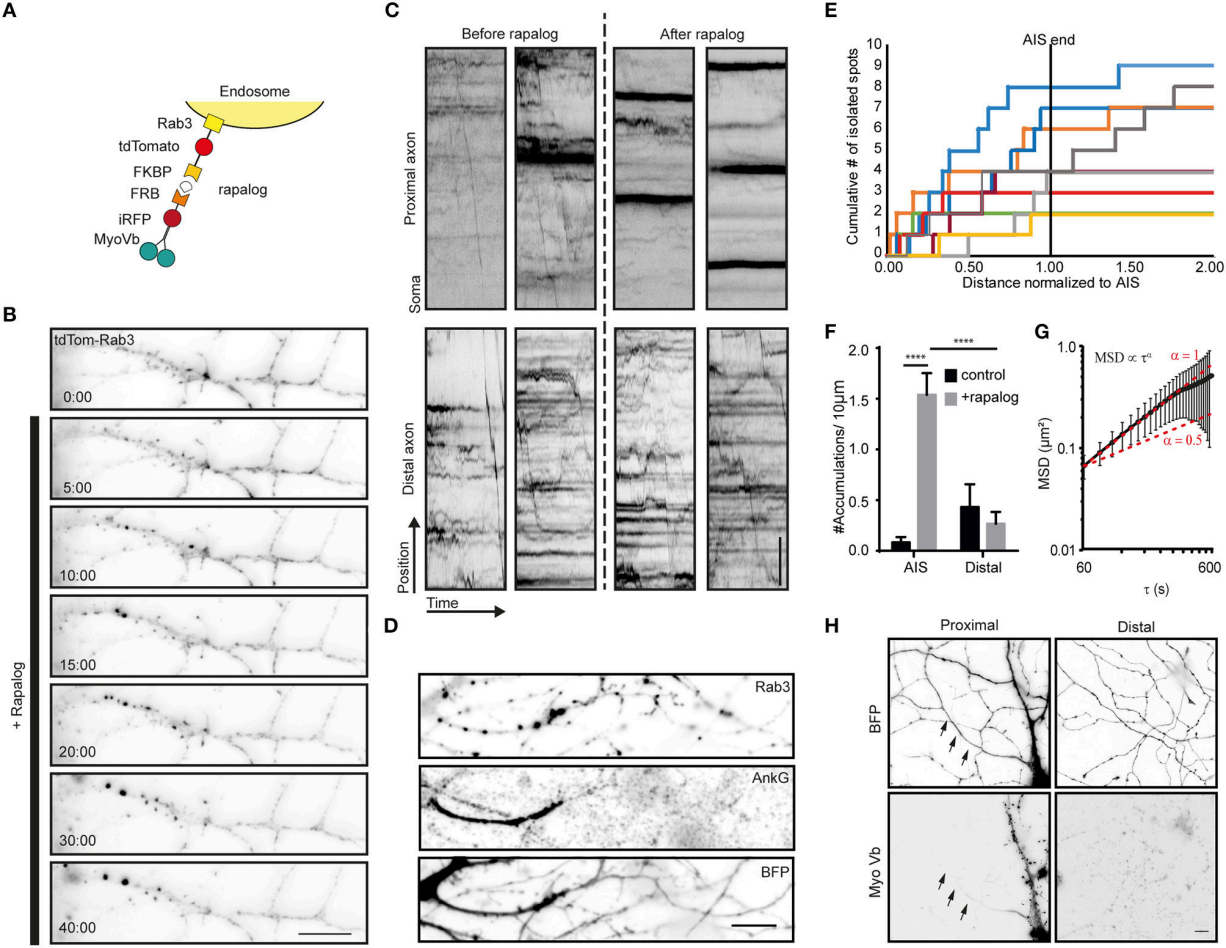
Myosin-V anchors Rab3 vesicles in the proximal axon of hippocampal neurons. **(A)** Assay: Recruitment of myosin-V to Rab3 vesicles by addition of rapalog. **(B)** Rab3 positive vesicle distribution in the proximal axon. Upon coupling of myosin-Vb to vesicles by addition of rapalog, Rab3 vesicles start accumulating in big puncta. Scale bar, 10 μm. **(C)** Kymographs of Rab3 vesicles in the proximal and distal axon. Short timelapses were acquired with 500 ms intervals before and after addition of rapalog. For imaging of distal axons after rapalog treatment, cells were chosen 1 h after rapalog addition that showed clear Rab3 anchoring in their proximal axon. Scale bar, 5 μm. **(D)** Rab3 distribution after myosin-Vb recruitment, together with a staining for Ankyrin-G to indicate the AIS. BFP was used as a fill to show the overall morphology. Scale bar, 10 μm. **(E)** Plot of the cumulative number of Rab3 accumulations found in the axon normalized to the AIS determined for cells after rapalog addition to recruit myosin-Vb. **(F)** Number of accumulations found in the AIS and the distal axon as determined using Ankyrin-G staining. The number of accumulation were determined in fixed cells with and without addition of rapalog Mean ± *sd* (*n* = 9 for both conditions), 2-way ANOVA reveals F_interaction_ = 26.27, *p* = 0.0001. *Post-hoc* multiple comparison testing: ^****^p < 0.0001. **(G)** Mean square displacement analysis of myosin-V anchored peroxisome clusters tracked for at least 25 intervals of 20 s. Mean ± *sd* (*n* = 18). **(H)** Distribution of the MyoVb(1-1090)-EGFP-FRB construct. The axon is indicated by arrows. Scale bar, 10 μm.

To explore how myosin-V affects the motility of cargoes that autonomously travel into the axon, we next turned to Rab3-positive vesicles. When myosin-Vb was recruited to Rab3 vesicles using the FKBP-rapalog-FRB system (Figure 3A), we observed the emergence of immobile clusters of Rab3-positive vesicles in the first part of the axon, whereas the motility of vesicles in the distal axon did not appear affected, based on inspection of the kymographs (Figures 3B,C; Supplemental Video 3). In addition, motility arrest and clustering was observed in the somatodendritic compartment (data not shown). Rab3 vesicle clustering was not observed upon treatment with rapalog or gibberellin in the absence of MyoVb-iRFP-FRB expression or without addition of rapalog in the presence of the motor (Supplemental Figures 2A,B). To test whether clustering was selective for the AIS, we repeated the experiment, followed by staining for the AIS marker Ankyrin-G (Figures 3D,E). Quantitative analysis of the Rab3 cluster relative to the staining of Ankyrin-G revealed that 92 ± 17% of clusters were found in AIS (average ± *sd, n* = 9 cells; Figure 3F). MSD analysis revealed an anomalous diffusion exponent of ~1 for time scales <360 s (Figure 3G). At longer times, the MSD leveled off, suggesting that Rab3 cluster motility was confined to 700–800 nm [i.e., (0.6 μm^2^)^1/2^]. It is important to note that the selective clustering in the AIS could be a trivial consequence of the very low levels of myosin-V in the remainder of the axon (Figure 3H). Nevertheless, these results demonstrate that myosin-V induces cargo clustering, rather than retrograde transport.

**Figure 4:**
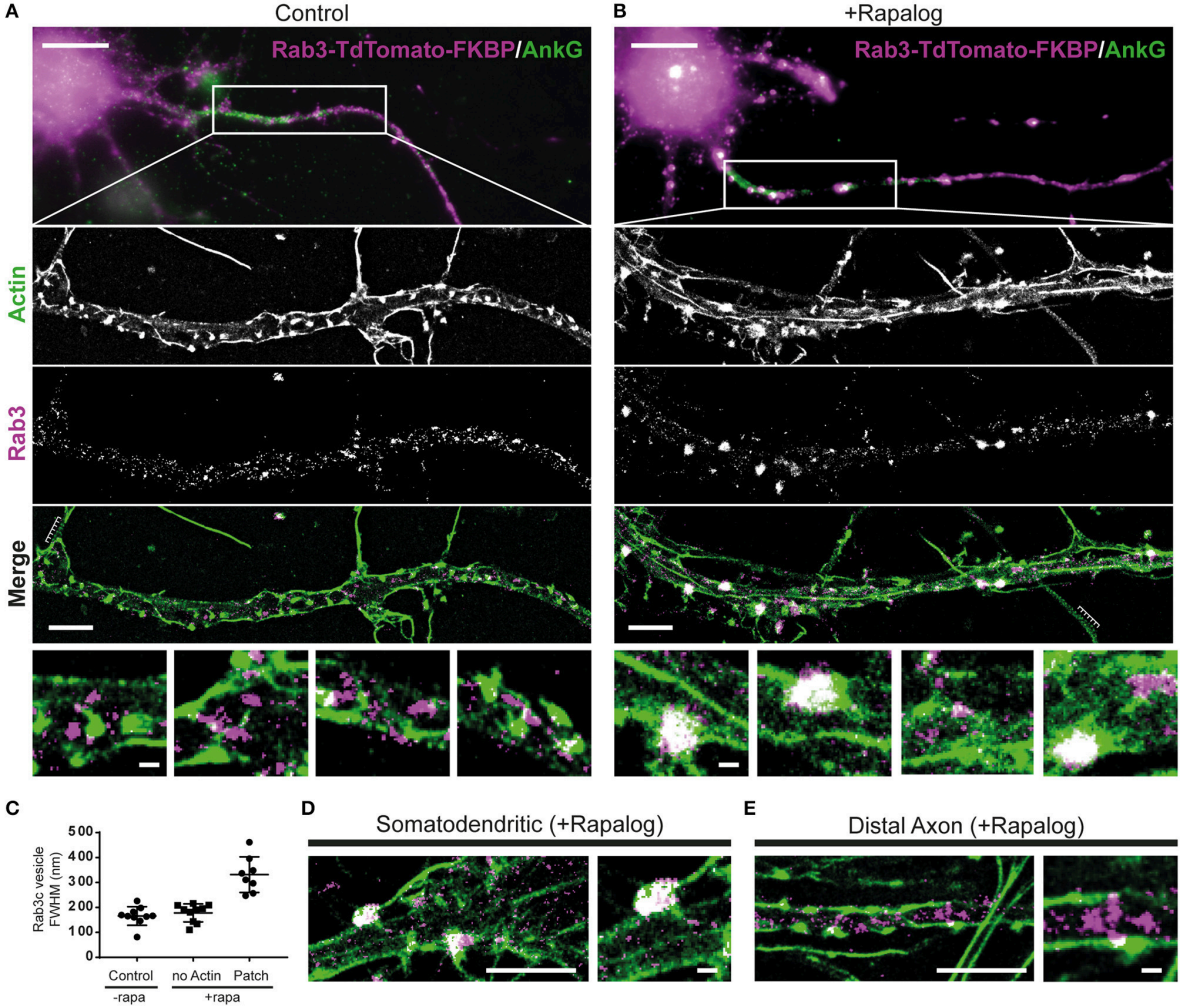
Myosin-V-anchored Rab-3 vesicles accumulate on actin patches in the proximal axon. **(A,B)** Single molecule localization microscopy of sequentially imaged lifeAct-GFP protein-PAINT and DNA-PAINT to visualize actin and Rab3, respectively. DIV10 neurons overexpressing MyosinVb-HA-FRB and Rab3-tdTomato-FKBP, post-stained for TdTomato and AnkyrinG. Axons were identified by AnkyrinG staining. Super-resolution images are shown for the regions indicated in the widefield images (top panel). Control **(A)** and MyosinVb coupled Rab3 vesicles **(B)** are imaged together with actin (middle 3 panels). Scale bar: top panel 10 μm, middle panels 2 μm, zooms 0.2 μm. **(C)** Quantification of the FWHM of Rab3 structures in control and rapalog-treated cells. Myosin-coupled Rab3 vesicles were separated based on their colocalization with actin patches. Mean ± *sd* are depicted (*n* = 8–10 for 2 cells per condition). **(D)** Super-resolution image and zoom of Rab3 cluster colocalization with actin in the somatodendritic compartment. Scale bar: left panel 2 μm, right panel 0.2 μm. **(E)** Super resolution image and zoom of Rab3 vesicles and actin in the distal axon. Scale bar: left panel 2 μm, right panel 0.2 μm.

Recent work has suggested a role for specialized actin structures in the AIS in myosin-V based cargo retrieval (Watanabe et al., 2012). To examine the relation between myosin-V induced cargo clustering and the actin cytoskeleton, we next performed superresolution microscopy to image actin and Rab3 in MyoVb-GFP-FRB and FKBP-tdTomato-Rab3c expressing neurons (Figures 4A,B). To visualize actin, we purified GFP-tagged Lifeact, a small probe that transiently interacts with polymerized actin and can be used to achieve the repetitive low density labeling required for single molecule localization microscopy (Kiuchi et al., 2015). To visualize Rab3, we used DNA-PAINT, in which a secondary antibody is labeled with an oligonucleotide that can transiently hybridize with a fluorescently labeled complementary strand, which also ensures repetitive low density labeling (Jungmann et al., 2014). After optimization of the extraction and fixation protocols, this enables us to perform two-color nanoscopy of the actin network and rab3 vesicles.

In control cells that were not treated with rapalog, we observed both regularly spaced actin stripes, as described previously (Xu et al., 2013), long actin fibers and distinct actin patches with concentrated staining (Figure 4A). No apparent colocalization between actin and Rab3 was observed in these cells. In cells treated with rapalog, a clear colocalization between Rab3 accumulations and actin-rich regions was observed (Figure 4B). The diameter of Rab3 structures that were colocalized with actin patches was about two times greater compared to Rab3 structures outside these patches or in cells without rapalog treatment (Figure 4C). This suggests that multiple vesicles have coalesced on these patches, consistent with our live imaging data of cluster formation (Figures 3B,C). Furthermore, Rab3 clusters were only observed in the proximal and not the distal axon (Figures 4D,E). These data demonstrate that actin patches are the site of myosin-mediated anchoring.

## Discussion

We have developed an assay for the sequential recruitment of motor proteins to specific cargoes. Using this assay, we were able to show that recruiting myosin-V to kinesin-1-driven cargo is sufficient for myosin to attenuate kinesin-driven motility of peroxisomes in COS7 cells. Future work will be directed toward exploring the myosin-kinesin ratio at which stalling still occurs. In addition, the outcome of other motor combinations could also be explored, for example kinesins and dyneins or combinations of different kinesin motors.

We were able to show a similar anchoring behavior in neurons where peroxisomes coupled to kinesin entered the axon, but were subsequently anchored at the AIS by myosin-V recruitment. Similar results were obtained upon recruitment of myosin-V to Rab3-positive vesicles. No reversals of myosin-V anchored peroxisomes back into the cell soma were observed. These results support a model in which myosin-V stalls the motility of dendritic vesicles that erroneously entered the axon, but also demonstrate that recruitment of myosin-V is not sufficient to bring these cargoes back into the soma to facilitate delivery to their proper destination. These finding are consistent with earlier work demonstrating that the coupling of a myosin-Va binding domain of Melanophilin to vesicles with no specific localization increases their halting frequency in the AIS but not the frequency of reversals (Al-Bassam et al., 2012).

Myosin-V-induced anchoring was also observed in the dendrites and soma, suggesting that this anchoring does not depend on specific feature of the AIS, but will occur whenever cargoes with active myosin-V enter actin-rich regions. Indeed, actin hotspots in the distal axon were also described by others (Ganguly et al., 2015). We do not exclude that recruitment of other proteins could also affect the transport of cargoes, for example due to steric effects. However, myosin-5 is recruited to kinesin that is already attached to an organelle and therefore the increase in size is not as dramatic compared to recruiting myosin-5 to a free kinesin. More importantly, myosin-5 recruitment stalls transport on actin patches, which suggests that this effect is at least specific to an actin-binding protein. Although, myosin-V based anchoring is not restricted to the axon, the actin in the AIS still establishes an important vesicle filter, because it enables the halting of cargo that is not supposed to enter the axon. This suggests that the cargo recruitment and/or activation state of myosin-V determines whether the cargo is allowed to pass the AIS. Upon anchoring, the subsequent recruitment of dynein could return the cargo to the cell body. This is consistent with recent work on the dynein regulator NDEL, which was shown to localize to the AIS via an interaction with the scaffolding protein Ankyrin-G and facilite cargo reversal (Kuijpers et al., 2016).

## Experimental procedures

### DNA constructs and protein purification

DNA constructs used in this study were cloned in pGW1-CMV and pβactin-16-pl vectors. The pβactin-PEX3-mRFP-FKBP-linker-GID1 construct was made by PCR amplification of the GID1 domain with addition of a linker (SAGGSAGGSAGG), then ligated into the SpeI and NotI sites of the pβactin-PEX3-mRFP vector described previously (Kapitein et al., 2010b), followed by PCR amplification of FKBP(1x) and insertion into the EcoRI and SalI sites of the construct. The FKBP encoding fragments were described previously (Kapitein et al., 2010b).

pβactin-MyoVb-(amino acid 1-1090)-GFP-FRB was described before (Kapitein et al., 2013). MyoVb-(1-1090)-iRFP-FRB (myosin-Vb) was generated by replacing the GFP by iRFP using the EcoRI and SpeI sites. Kif17md-GFP-GAI was generated by insertion of Kif17MD (aa 1-547 of human KIF17) in AscI and SalI sites, GFP in SalI and SpeI sites, and GAI(1-92) in SpeI and NotI sites of pβactin. FKBP-tdTomato-Rab3c was generated by insertion of PCR-amplified tdTomato in SalI and SpeI site, Mouse Rab3c in SpeI and NotI sites and FKBP(1x) in BamHI and SalI sites of the pβactin-16-pl vector.

To visualize actin using single molecule localization microscopy lifeAct-GFP was purified and used as a transient binding probe (Kiuchi et al., 2015). In brief, lifeAct- GS linker -GFP was cloned into a PET28a vector with a C-terminal 6x His sequence and transformed into BL21DE3 bacteria. Bacteria were grown until OD0.6 and induced with 1 mM IPTG overnight at 17°C. Cells were then pelleted and resuspended in resuspension buffer [20 mM HNa2PO4, 300 mM NaCl, 0.5% glycerol, 7% glucose, EDTA-free protease inhibitor (Roche Diagnostics GmbH), 1 mM dithiothreitol (DTT), pH 7.4]. Cells were lysed by sonication and the soluble and insoluble fraction were separated by centrifugation. Ni-NTA (Roche) beads were washed with resuspension buffer and incubated with the soluble supernatant for 1.5 h at 4°C. After incubation, the beads with bound proteins were washed 5 times with wash buffer (10 mM HNa2PO4, 300 mM NaCl, 30 mM imidazole, 1 mM DTT, pH 7.4). Finally lifeAct-GFP was eluted in Elution Buffer (10 mM HNa2PO4, 300 mM NaCl, 300 mM imidazole, 1 mM DTT, pH 7.4) and snap-frozen at −80°C with 10% glycerol.

### Cell cultures and transfection

COS-7 cells were cultured in DMEM/Ham's F10 (1:1) medium containing 10% FCS and penicillin/streptomycin. Cells were plated on 18-mm diameter coverslips 2–4 days before transfection. Cells were transfected with Fugene6 transfection reagent (Roche) according to the manufacturer's protocol and imaged 1 day after transfection.

Primary hippocampal cultures were prepared from embryonic day 18 (E18) rat brains (Kapitein et al., 2010c). Cells were plated on coverslips coated with poly-L-lysine (30 μg ml^−1^) and laminin (2 μg ml^−1^). Hippocampal cultures were grown in Neurobasal medium (NB) supplemented with B27 (Invitrogen), 0.5 mM glutamine, 12.5 μM glutamate, and penicillin plus streptomycin. Transfections of hippocampal neurons were performed 48 h before imaging with lipofectamine 2000 (Invitrogen). DNA (1.8 μg per well) was mixed with 3.3 μl lipofectamine 2000 in 200 ml NB, incubated for 30 min, and added to the neurons in NB supplemented with 0.5 mM glutamine at 37°C in 5% CO_2_. After 60–90 min neurons were washed with NB and transferred to the original medium at 37°C in 5% CO_2_ for 2 days. Transport assays in neurons were imaged at day-*in-vitro* 12–16.

### Live-cell imaging

Time-lapse live-cell imaging of peroxisomes in hippocampal neurons was performed on a Nikon Eclipse TE2000E (Nikon) equipped with an incubation chamber (Tokai Hit; INUG2-ZILCS-H2) mounted on a motorized stage (Prior; Kapitein et al., 2013). Coverslips (18 mm) were mounted in metal rings covered with conditioned medium. Cells were imaged every 30 s for 60 min using a 40 × objective [Plan Fluor, numerical aperture (NA) 1.3, Nikon] and a Coolsnap HQ2 CCD camera (Photometrics).

Peroxisomes in COS7 and neurons were images using a 40x objective (Plan Fluor, numerical aperture (NA) 1.3, Nikon) in Ringer's solution (10 mM HEPES, 155 mM NaCl, 5 mM KCl, 1 mM CaCl_2_, 1 mM MgCl_2_, 2 mM NaH_2_PO_4_, and 10 mM glucose, pH 7.4) or conditioned culture medium, respectively. Rab3 vesicles in neurons were imaged in conditioned medium using a 100 × objective (Apo TIRF, 1.49 NA, Nikon). A mercury lamp (Osram) and filter wheel containing ET-GFP (49002), ET-dsRed (49005), ET-mCherry (49008), and ET-GFPmCherry (59022) emission filters (all Chroma) were used for excitation. Rab3 in neurons were imaged on a CoolSNAP MYO CCD camera (Photometrics) and peroxisomes in neurons and COS7 cells with a Coolsnap HQ camera (Photometrics, Tucson, AZ). During imaging, all cells were maintained at 37°C, as well as 5% CO_2_ when using conditioned medium.

Cell-permeable gibberellin (GA3IAM, a gift from Dr. T. Inoue; Miyamoto et al., 2012) and Rapalog (AP21967 from Ariad Pharmaceuticals) were added during image acquisition to reach a final concentration of 150–300 and 100 nM, respectively at the indicated time points. In hippocampal neurons, the axon was identified based on morphology and Rab3 vesicle enrichment. The proximal axon was defined as the first part of the axon before branching, whereas distal axon refers to axonal segments after at least 2 branch points.

To identify the axon in live cell imaging experiments (Supplemental Figure 1), neurons were stained for extracellular Neurofascin before imaging. Coverslips were placed in cultured medium containing anti-neurofascin (Neuromab, mouse, 1/200) for 10 min. Coverslips where then washed 5 times by briefly dipping them in neurobasal. Subsequently, coverslips were incubated with anti-mouse AlexaFluor405 (Life Technologies, anti-mouse, 1/100) for 10 min. After 5 additional washes cells were place back into their cultured medium before imaging.

### Cell fixation and single molecule localization imaging

Cells transfected with Rab3-TdTomato-FKBP, MyosinVb-HA-FRB, and a BFP fill (Figures 3D–H) were fixed with 4% PFA in PBS for 10 min at 37°C. Subsequently, cells were washed 2 times with PBS, permeabilized with 0.25% triton in PBS for 10 min and washed again 3 times with PBS. After washing, cells were blocked for 45 min in blocking solution (2% w/v BSA, 0.2% w/v gelatin, 10 mM glycine, 50 mM NH4Cl in PBS, pH 7.4) and incubated overnight with anti-Ankyrin-G (1/200, mouse, Life technologies). Cells were further incubated with anti-mouse Alexa647 (1/400, Life technologies) and mounted for imaging in mowiol.

To determine the localization of the Rab3 accumulation (Figure 3) samples were imaged on a Nikon eclipse TI upright microscope with a 40x objective (UPLFLN, NA 1.3). Myosin-Vb localization was imaged on an Olympus BX53 upright microscope with a 60x objective (oil, UPLSAPO, NA1.35).

For simultaneous super-resolution imaging of Rab3 and actin (Figure 4) cells overexpressing Rab3-TdTomato-FKBP and MyosinVb-HA-FRB (either treated with rapalog or not), were pre-extracted 1 min with 0.35% glutaraldehyde and 0.25% triton-x in cytoskeleton buffer (Xu et al., 2013). Cells were then further fixed with 4% PFA. Subsequently, samples were washed 3x with PBS followed by 10 min permeabilization in PBS + 0.25% triton-x. After 3 more 5 min washes in PBS, samples were blocked in 3% BSA for 45 min followed by overnight 4°C staining with anti-AnkG (1/200, mouse, Life technologies) and anti-RFP (1/500, rabbit, Rockland) in blocking buffer. After incubation cells were washed 3 times 10 min in PBS and incubated with secondary anti-mouse AlexaFluor488 (1/400, Life Technologies) and anti-rabbit-D2 from the Ultivue-2 super resolution 2-plex kit (1/100, Ultivue) for 1.5 h at room temperature in blocking buffer. After 3 additional washes samples were mounted in a Ludin chamber in Imaging Buffer (Ultivue). Single-molecule microscopy was performed on a Nikon Ti-E microscope equipped with a 100x Apo TIRF oil immersion objective (NA. 1.49) and Perfect Focus System 3. Excitation was achieved via a custom illumination pathway with a Lighthub-6 (Omicron) containing a 638 nm laser (BrixX 500 mW multimode, Omicron), a 488 nm laser (Luxx 200 mW, Omicron), and a 405 nm laser (Luxx 60 mW, Omicron). Emission light was separated from excitation light with a quad-band polychroic mirror (ZT405/488/561/640 rpc, Chroma), a quad-band emission filter (ZET405/488/561/640 m, Chroma), and an additional single-band emission filter (ET525/50 m for green emission and ET655lp for far-red emission, Chroma). Fluorescence was detected using a sCMOS camera (Hamamatsu Flash 4.0v2). Samples were positioned in the *x*- and y-direction with an M-687 PILine stage (PI).

For super-resolution imaging, first cells expressing Rab3-TdTomato were selected and AnkyrinG was imaged to identify the axon. LifeAct-GFP and Imager strand I2-650 (Ultivue) were diluted so that single molecule binding events could be observed for both channels. Subsequently, the relatively weak AnkyrinG AlexaFluor488 staining was completely bleached and LifeAct based protein-PAINT (Kiuchi et al., 2015) was performed by observing single binding and unbinding effects. Subsequently, DNA-PAINT was performed similarly for Rab3 structures stained with rabbit-D2 and Imager strand I-2. For both channels between 8,000 and 15,000 frames were acquired with a 100 ms exposure time to reconstruct super resolved images of both actin and Rab3. Images were then reconstructed using our ImageJ plugin called DoM (Detection of Molecules, https://github.com/ekatrukha/DoM_Utrecht) which has previously been described in detail (Yau et al., 2014; Chazeau et al., 2016).

### Image processing and analysis

Images of live cells were processed and analyzed using MetaMorph (Molecular Devices), LabVIEW (National Instruments) software and ImageJ (NIH). Drift correction was applied using the StackReg plugin for ImageJ (Thevenaz et al., 1998) for time series during which multiple positions were recorded using a motorized stage.

To generate the radial kymograph, pixels that were above the set threshold were inserted into a histogram representing the intensity vs. the distance from the center of the cell. This was done for each video frame using the camera pixel size as bin size (Kapitein et al., 2010b).

For analysis of redistribution dynamics in COS7 cells, cells were masked to exclude contributions from neighboring cells to the analysis. A threshold was set for all images of a time-lapse recording at ~6–12 times the standard deviation of the background above the background to yield binary images. These thresholds were set manually such that individual peroxisomes were suprathreshold (by an experimenter who was not blind to experimental group). The same thresholds were set for analysis of the *R*_90%_ and calculations of the correlation index. To quantify peroxisome redistribution upon recruitment of motor proteins in COS7 cells, the radius required to include 90% of the total intensity of the cell, *R*_90%_(*t*), was calculated for each frame as described previously (Kapitein et al., 2010b). To quantify changes in the dynamics of peroxisomes upon recruitment of (additional) motor proteins, we calculated the time-dependent frame-to-frame correlation index*c*_τ_(*t*) as described before (van Bergeijk et al., 2015). A value of 1 for *c*_τ_(*t*) indicates that particles are completely anchored and thus their position is unchanged after a time τ, whereas a value of 0 means that all particles moved to locations that were previously unoccupied. For statistical analysis on R90% and correlation index, average values of 10 frames at *t* = 0–4.5, 25–29.5, and 55–59.5 min were used. Friedman test was performed with Dunn's *post-hoc* test.

To quantify the movement of the peroxisome or Rab3 accumulations formed after recruiting Myosin-V in the proximal axon (Figures 2E, 3G), their positions were analyzed between 10 and 25 min after the addition of rapalog with 30 s interval acquisition. The spots were tracked using the trackmate plugin for ImageJ. For every time point the x-position relative to the initial position was plotted. For these trajectories, mean square displacement analysis was performed using the MSDanalyzer (Tarantino et al., 2014) class for MATLAB, including tracks that were at least 25 time points long (*n* = 26 for Figure 2E and *n* = 18 for Figure 3G).

To analyze Rab3 clustering (Figures 3E,F), the number of bright isolated spots as shown in Figure 3D were compared between the proximal axon (colocalizing with the AIS marker Ankyrin-G, which marked a segment with visibly identifiable boundaries) and more distal segments (further than the second branch), both in the presence and absence of rapalog. For the quantification of the Rab3 accumulations relative to the AIS, the AIS length was measured manually based on the bright Ankyrin-G staining that defines the AIS. Subsequently, the location of the rab3 accumulation was divided by the measured length of the AIS.

## Author contributions

LK conceived research. PvB, RO, and AJ designed, created, and tested constructs. AJ and RT performed experiments and analyzed data. CH provided conceptual input and provided neuron cultures. AJ, RT, and LK wrote the paper with input from all other authors. LK supervised research.

### Conflict of interest statement

The authors declare that the research was conducted in the absence of any commercial or financial relationships that could be construed as a potential conflict of interest.

## Supplementary material

Supplemental Figure 1 Corresponds to Figure 2. **(A)** GFP fill (left) and live neurofascin staining (middle) of a cell expressing GFP, Pex3-mRFP-FKBP, and Kif5-GFP-FRB. **(B)** Partial maximum projections of the peroxisome-redistribution experiment performed on the cell in **(A)**, before (left) and after rapalog addition (right). Red arrows indicate the axon identified by neurofascin. **(C)** Percentage of cells with peroxisome targeting into the axon or both axon and dendrite, after recruitment of kif5-GFP-FRB. In all cases the axon was identified by the live neurofascin staining as in **(A,B)**. *n* = 21.

Supplemental Figure 2 Corresponds to Figure 3. **(A)** Rab3 positive vesicle distribution in the proximal axon of a neuron that co-expressed myosin-Vb, but was not treated with ligands (left panels), or did not express myosin Vb and was treated with Gibberellin (middle panels) or rapalog (right panels). In these control conditions Rab3 vesicles do not accumulate in big puncta as seen by selective recruitment of myosinVb. Scale bar, 10 μm. **(B)** Kymographs of Rab3 vesicles in the proximal axon of cells co-expressing myosin-Vb with and without addition of rapalog, or cells without myosinVb and treated with Gibberellin or Rapalog. Timelapse images were acquired with 20 s intervals. Arrows indicate Rab3 accumulations appearing after rapalog addition. Scale bar, 5 μm.

Supplemental Video 1 Sequential recruitment of kinesin and myosin-V in a COS7 cell. This video corresponds to Figure 1. Anchoring of kinesin propelled cargo by Myosin-V in a COS7 cell. Total time: 60 min. Acquired with 30 s between frames. 600x sped up.

Supplemental Video 2 Myosin-V anchors kinesin-1 propelled peroxisomes in the proximal axon. This video corresponds to Figure 2. Anchoring of kinesin propelled cargo by Myosin-V in a hippocampal neuron. Total time: 60 min. Acquired with 30 s between frames. 600x sped up.

Supplemental Video 3 Myosin-V anchors Rab3 vesicles in the AIS of hippocampal neurons. This video corresponds to Figure 3. Anchoring and clustering of Rab3 vesicles in the proximal axon of hippocampal neurons. Total time: 40 min. Acquired with 10 s between frames. 200x sped up.
