# Supplementary figures and images for: Myosin-V Induces Cargo Immobilization and Clustering at the Axon Initial Segment
Source: Front Cell Neurosci. 2017 Aug 28;11:260. doi: 10.3389/fncel.2017.00260 (PMC5581344; doi:10.3389/fncel.2017.00260)

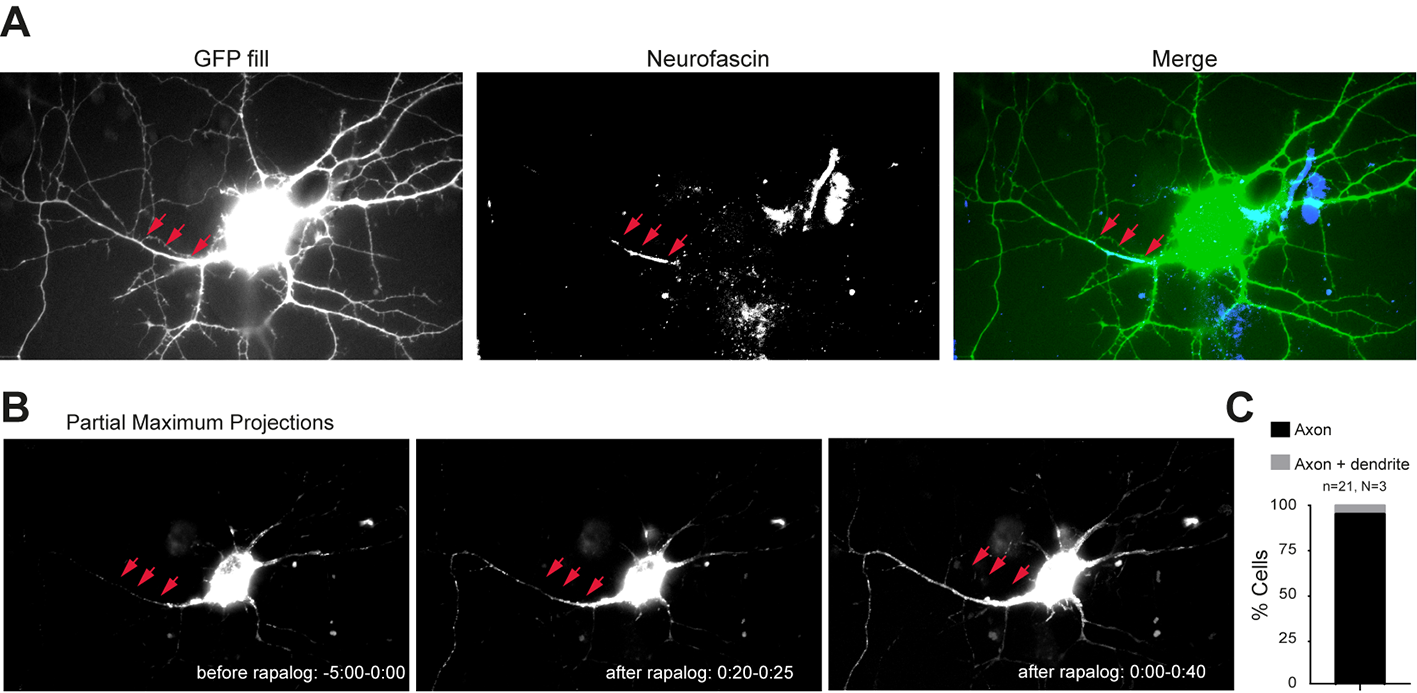

Supplement: Supplemental Figure 1 — Corresponds to Figure 2. (A) GFP fill (left) and live neurofascin staining (middle) of a cell expressing GFP, Pex3-mRFP-FKBP, and Kif5-GFP-FRB. (B) Partial maximum projections of the peroxisome-redistribution experiment performed on the cell in (A), before (left) and after rapalog addition (right). Red arrows indicate the axon identified by neurofascin. (C) Percentage of cells with peroxisome targeting into the axon or both axon and dendrite, after recruitment of kif5-GFP-FRB. In all cases the axon was identified by the live neurofascin staining as in (A,B). n = 21. [file Image1.tif]

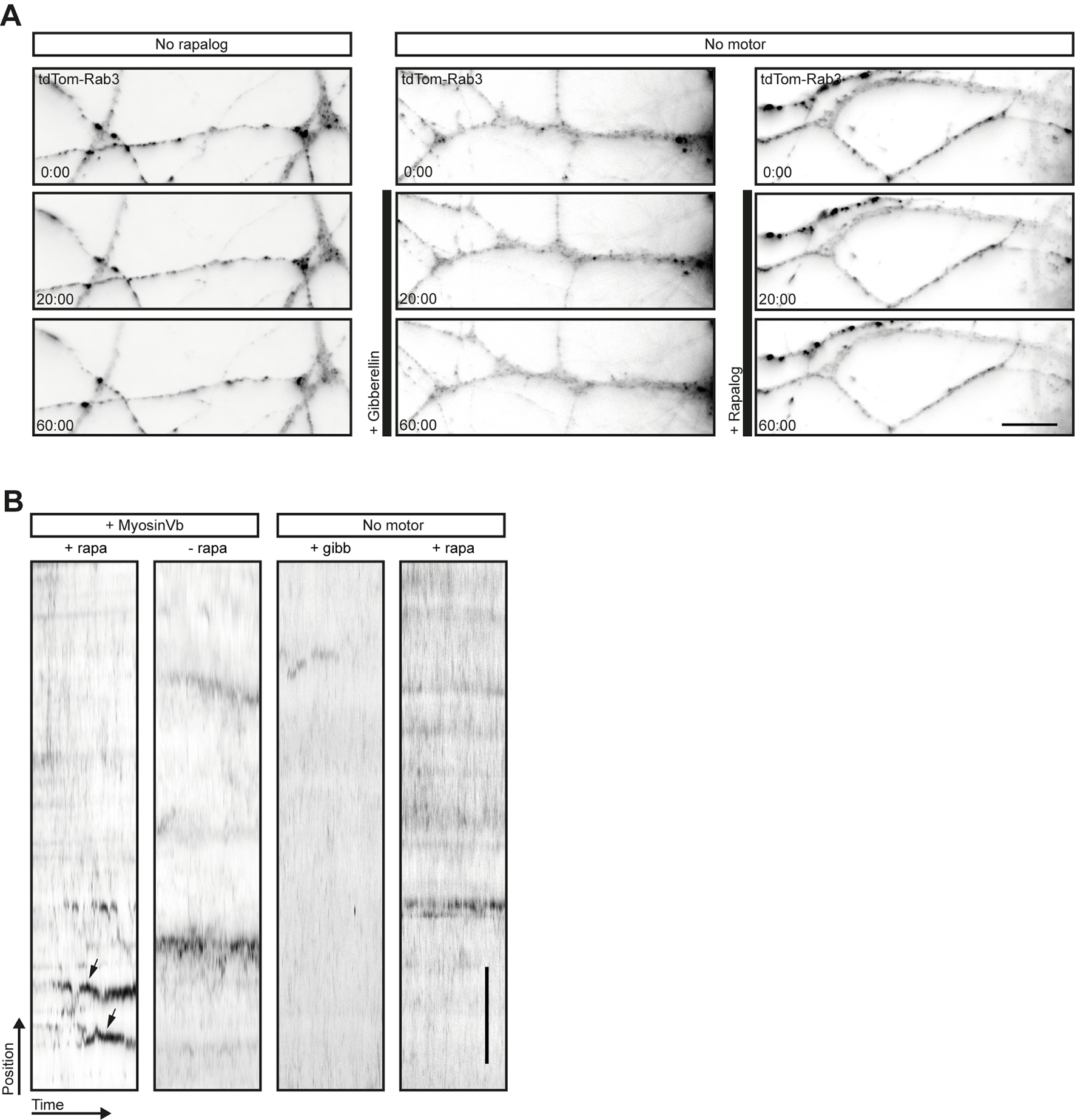

Supplement: Supplemental Figure 2 — Corresponds to Figure 3. (A) Rab3 positive vesicle distribution in the proximal axon of a neuron that co-expressed myosin-Vb, but was not treated with ligands (left panels), or did not express myosin Vb and was treated with Gibberellin (middle panels) or rapalog (right panels). In these control conditions Rab3 vesicles do not accumulate in big puncta as seen by selective recruitment of myosinVb. Scale bar, 10 μm. (B) Kymographs of Rab3 vesicles in the proximal axon of cells co-expressing myosin-Vb with and without addition of rapalog, or cells without myosinVb and treated with Gibberellin or Rapalog. Timelapse images were acquired with 20 s intervals. Arrows indicate Rab3 accumulations appearing after rapalog addition. Scale bar, 5 μm. [file Image2.TIF]
